# Gomisin J attenuates cerebral ischemia/reperfusion injury by inducing anti-apoptotic, anti-inflammatory, and antioxidant effects in rats

**DOI:** 10.1080/21655979.2022.2026709

**Published:** 2022-03-02

**Authors:** Xiaoli Min, Linping Zhao, Ying Shi, Jian Wang, Hongling Lv, Xiaoxiao Song, Qunyuan Zhao, Qing Zhao, Rui Jing, Jiayi Hu

**Affiliations:** a Department of Cerebrovascular Diseases, The Second Affiliated Hospital of Kunming Medical University, Kunming, Yunnan Province, China; b Department of Internal Medicine, Clinical Medicine School, Yunnan Traditional Chinese Medicine University, Kunming, Yunnan Province, China; c Yunnan Communications Vocational and Technical College, Institute of International Exchange, Kunming, Yunnan Province, China; d Department of Epidemiology and Statistics, Public Health School, Kunming Medical University, Kunming, Yunnan Province, China; e Department of Emergency, The Second Affiliated Hospital of Kunming Medical University, Kunming, Yunnan Province, China

**Keywords:** Cerebral ischemia-reperfusion injury, stroke, Gomisin J, apoptosis, inflammation, oxidative stress

## Abstract

Ischemic stroke is one of the leading causes of morbidity and mortality in humans. Cerebral ischemia-reperfusion (CIR) injury serves as a leading cause of stroke. *Schisandra chinensis* is a well-known Chinese traditional medicine. In this study, we explored the role of Gomisin J (GJ), a compound of *S. chinensis*, in CIR using a middle cerebral artery occlusion/reperfusion rat model and the possible mechanisms. We identified that GJ reduced neurological scores, cerebral infarction, and water content in the I/R rat brain. Importantly, GJ rescued I/R treatment-reduced neuron survival in the hippocampus, inhibited apoptosis of ischemic tissues in I/R rats, increased B-cell lymphoma-extra-large (Bcl-XL) expression, and reduced the levels of cleaved caspase-3, Bax, cyclooxygenase-2, nuclear factor kappa-B, and nitric oxide in I/R rat brain tissues. Furthermore, GJ treatment enhanced nuclear factor E2 related factor 2 (Nrf2) translocation, heme oxygenase-1 (HO-1) expression, superoxide dismutase and glutathione peroxidase activities, and glutathione level. Overall, GJ treatment GJ attenuates CIR injury by inducing anti-apoptotic, antioxidant, and anti-inflammatory effects *in vivo*.

## 1. Introduction

Stroke, a general cause of long-course of serious disorders and mortality globally [1,2], is usually provoked by cerebral ischemia due to embolism occlusion of prominent brain aorta or thromboembolism [3]. To intervene stroke, reperfusion is often performed for blood restoration. However, the procedure easily leads to cerebral ischemia/reperfusion (CIR) injury [4] due to hypoxia following the short-course restoration of blood reperfusion [5]. Increasing studies have indicated that CIR injury usually comprises neural phenomena, such as inflammatory response, oxidative stress, and hypoxia [6], which eventually results in necrosis, apoptosis, and acute autophagy in the ischemic cerebrum [7]. Currently, tissue plasminogen activator (tPA) is the only practical approach to manage CIR injury [8]. Therefore, it is urgent and necessary to recognize novel and useful therapeutic targets and develop effective agents for CIR injury treatment for patients with stroke.

In recent years, various novel traditional Chinese medicines have been formulated to treat cerebrovascular disorders [9–11]. For example, *Radix Scrophulariae* has been identified to attenuate CIR injury by modulating MAPK signaling [12]. *Schisandra Chinensis* is a wood herb native to China, Japan, and Korea [13]. *S. Chinensis* has been recognized as a common herb medicine to treat and alleviate many disorders [14,15]. It comprises various active chemical components, including gomisins, pregomisin, deoxyschisandrin, schisandrin, and lignans and is involved in many signaling pathways to modulate various biological processes and exert various biological roles [16–18], such as anti-cancer, obesity, adipogenesis, oxidative stress, inflammation, fibrosis, and vascular contractility. Gomisin J (GJ) is a major component of *S. Chinensis* and has been identified to present numerous biological activities, including antiretroviral activity, anti-inflammation, defensive impact on angiotensin II-related hypertension, anti-hepatic effects on cardiovascular symptoms, and vascular relaxation [19–21]. All these imply that GJ may possess potential functions in modulating cerebrovascular disorders. However, the effect of GJ on CIR injury is still elusive. Therefore, we constructed a middle cerebral artery occlusion/reperfusion (MCAO/R) rat model and explored the role of GJ in CIR injury. Our data showed that GJ relieved neuronal injury, attenuated neurological loss of the hippocampus, repressed apoptosis of ischemic tissues, induced anti-inflammatory and antioxidant effects, and decreased lipid peroxidation. Our results indicated that GJ might perform a novel function in inhibiting CIR injury.

## 2. Materials and methods

### 2.1. Experimental animals and grouping

Male Wistar rats (n = 180, body weight 250–300 g) from Shandong Pengyue Experimental Animal Company were used in the study. They were kept under the restriction of a controlled temperature (25 ±1°C) and light cycles (12 h light/12 h dark) and allowed free access to water and food. Rats were randomly divided into sham (n = 18), I/R(n = 30), and I/R plus GJ groups. Rats in the I/R plus GJ group were further divided into 5 mg/kg GJ (n = 18), 10 mg/kg GJ group (n = 18), 20 mg/kg GJ group (n = 18), 40 mg/kg GJ group (n = 18), and 80 mg/kg GJ group (n = 30).

### 2.2. MCAO/R rat model

To analyze CIR injury, the MCAO/R model was constructed as previously reported [22]. Briefly, rats were placed in a fixed frame and quickly anesthetized using sodium pentobarbital (30 mg/kg). Then, the blood was occluded from the common carotid artery through external carotid artery by inserting a 4–0 nylon filament into the internal carotid artery for 2 h. After that, the blood flow was restored for 3, 6, 12, 24, or 48 h. Rats in the sham group received the same operation without MCAO/R occlusion. Neurological function score was recorded after reperfusion, and brain tissues were obtained for subsequent analysis. All procedures and animal care were authorized by the Animal Ethics Committee of The Second Affiliated Hospital of Kunming Medical University.

### 2.3. Drug administration

GJ (purity ≥ 99%, MCE, USA) was dissolved in PBS with 0.1% dimethyl sulfoxide (DMSO) and 1% hydroxyethyl cellulose. GJ was intraperitoneally injected into rats at 5, 10, 20, 40, and 80 mg/kg body weights [23,24] in the I/R plus GJ group before cerebral reperfusion. Rats in the I/R and sham groups were intraperitoneally injected with PBS with 0.1% dimethyl sulfoxide (DMSO) and 1% hydroxyethyl cellulose.

### 2.4. Brain damage analysis

The neurological deficits of rats after MCAO/R operation were evaluated by behavioral inspection and scored from 0 to 5 points by standard criteria [25]. Rats without neurologic deficit were scored 0 point; Rats with mild focal neurologic deficit and failure to extend left forepaw fully were scored 1 point; Rats with moderate focal neurologic deficit and circling to the left were scored 2 points; Rats with severe focal deficit and falling to the left were scored 3 points; Rats with depressed consciousness and unable to walk spontaneously were scored 4 points; and rats that died during experiments were scored 5 points. 2,3,5-Triphenyltetrazolium chloride (TTC, Sigma, USA) was used to evaluate cerebral infarction. At 24 h after operation, 6 rats from each group were sacrificed, and the whole brains were rapidly removed. In addition, the infarct volumes of rats in the 80 mg/kg GJ and the I/R groups were estimated at 3, 6, 12, and 48 h after reperfusion. The brain samples were coronally sectioned into 2 μm slices, incubated with 2% TCC solution for 20 min, fixed using 4% paraformaldehyde, and observed under a microscope. The normal tissues were stained red with TCC, while the infarcted sections appeared white. The infarcted volume was calculated [26] as previously described. The brain water content (BWC) was determined by the difference in infarct hemisphere weight before and after being dried [12]. Hematoxylin and eosin staining was used to analyze the numbers of surviving neurons of the hippocampus in the rats.

### 2.5. TUNEL assay

Samples were fixed in 10% formalin for 24 h, dehydrated in gradient ethanol solutions, and prepared as 5 μm paraffin sections using a microtome. The sections were dewaxed, incubated with 0.1 M PBS containing 0.3% Triton X-100 for 30 min, and stained with TUNEL reagents (Roche, Germany) for 1 h. In addition, ventricular samples were dyed using DAPI (Sigma, USA) after TUNEL staining. TUNEL-positive cells were detected using a fluorescence microscope.

### 2.6. Lipid peroxidation analysis

Lipid peroxidation in the rats was analyzed by measuring the levels of malondialdehyde (MDA) using the thiobarbituric acid reaction. In short, rat brains were removed after reperfusion and homogenized. The tissue homogenates were incubated with 10% trichloroacetic acid (TCA) solution and 0.67% thiobarbituric acid (TBA) solution at 100°C for 30 min. The supernatants were transferred into 96-well plates, and the absorption at 532 nm was measured using a microplate reader. The MDA concentration was calculated based on a standard curve.

### 2.7. NO production analysis

NO production was analyzed using the Griess assays. In short, the brain tissues were entirely removed and homogenized in a saline solution on ice. The supernatants were reacted with a solution containing 0.1% N-1-naphthyl ethylenediamine dihydrochloride, 5% H_3_PO_4_, and 1% sulfanilamide at room temperature in 96-well plates in the dark. After that, the supernatants were transferred into 96-well plates, and absorptions at 532 nm were measured using a microplate reader (BioTek Instruments, USA).

### 2.8. Activity measurement of antioxidant enzymes

Brain tissues were removed and homogenized in a saline solution on ice. Superoxide dismutase (SOD) activity, glutathione (GSH) content, and glutathione peroxidase (GSH-Px) activity were determined using a SOD assay kit, a GSH assay kit (Cayman Company, USA), and a GSH-Px assay kit (Cayman Company, USA), respectively, by measuring absorptions at 450 nm, 405 nm, and 340 nm, respectively, with a microplate reader (BioTek Instruments, Winooski, USA).

### 2.9. Western blot analysis

Total proteins were extracted, quantified using a BCA kit, separated by SDS-PAGE, and transferred onto PVDF membranes. The membranes were incubated overnight with primary antibodies against caspase-3, Bax, and Bcl-2 at dilution 1:1000 from Cell Signaling Technology, USA, and p-p65, COX-2, Nrf2, Lamin B1, HO-1 and β-actin at dilution 1:1000 from Abcam, USA. The membranes were then incubated with proper second antibodies for 1 h. The signals were visualized using Odyssey CLx Infrared Imaging System and quantified using ImageJ software.

### 2.10. Statistical analysis

Data were expressed as mean ± SD and quantitatively analyzed using Graphpad prism 7. The neurobehavioral scores were analyzed using a non-parametric (Mann-Whitney) U test. Differences among multiple groups and between two groups were analyzed using one-way ANOVA and unpaired Student’s t-test, respectively. *P* < 0.05 were considered statistically significant.

## 3. Results

The study explored whether GJ, a compound of *S. chinensis*, plays a role in attenuating CIR injury in the middle cerebral artery occlusion/reperfusion rat model. We found that GJ relieved neuronal injury, attenuated neurological loss of the hippocampus, repressed apoptosis of ischemic tissues, exerted an anti-inflammatory and antioxidant effect, and inhibited I/R injury-induced lipid peroxidation.

### 3.1. GJ relieves neuronal injury in I/R rats

**Figure 1.**
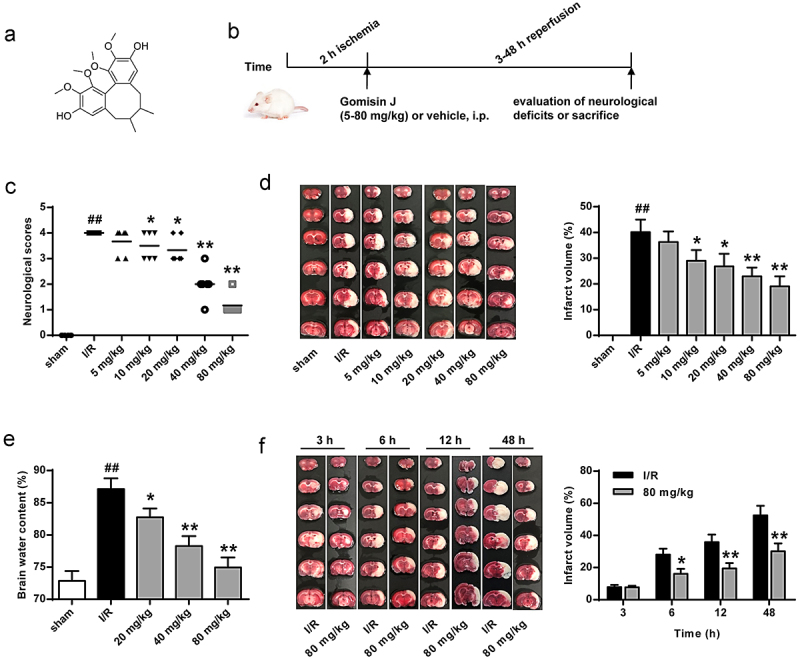
GJ relieves I/R rat neuronal injury. (a) GJ chemical structure. (b) Experimental protocol schematic diagram. (c-f) MCAO/R rats were treated with GJ at indicated doses (n = 6). (c) Rat neurological deficit scores were measured after 24 h of reperfusion. (d) TC staining of cerebral infarction in rats. (e) Rat brain water content was calculated after 24 h of reperfusion. (f) TC staining of cerebral infarction after reperfusion in the rats. ** *P* < 0.01, ^#^ *P* < 0.05, ^##^ *P* < 0.01. Data are presented as mean ± SD.

To understand GJ’s role in modulating CIR injury, we constructed a MCAO/R rat model. Figure 1 shows the GJ structure (Figure 1(a)) and experimental flowchart (Figure 1(b)). The neurological scores of rats were significantly increased by the I/R treatment at 24 hours after reperfusion, and GJ treatment dose-dependently reduced this increase (*P* < 0.01, Figure 1(c)). Meanwhile, TTC staining showed that GJ treatment inhibited I/R-induced cerebral infarction (*P* < 0.01, Figure 1(d)) and reduced brain water content (*P* < 0.01, Figure 1(e)) in rats in a dose-dependent manner. Furthermore, we found that 80 mg/kg GJ treatment decreased the infarct volume in I/R rats at 6 h, 12 h, and 48 h of reperfusion. Altogether, these suggest that GJ relieves neuronal injury in I/R rats.

### 3.2. GJ attenuates neurological loss in I/R rat hippocampus

**Figure 2.**
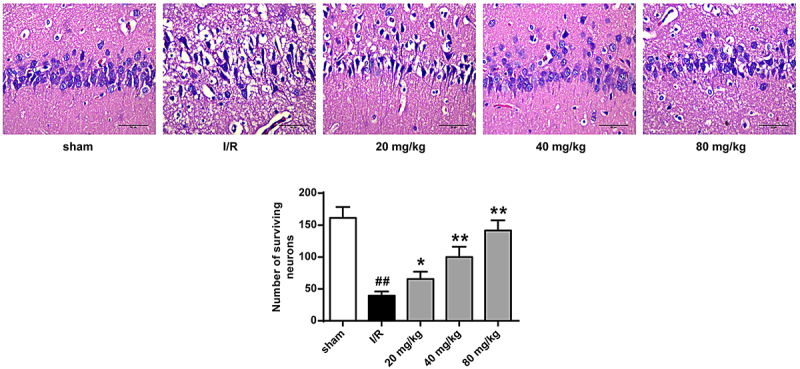
GJ attenuates the neurological loss of hippocampus in I/R rats. MCAO/R rat model was constructed and treated with GJ at indicated doses (n = 6). Hematoxylin & Eosin staining of hippocampus neurons in the rats (200×). * *P* < 0.05, ** *P* < 0.01, ^##^ *P* < 0.01. Data are presented as mean ± SD.

Next, we further explored GJ’s neuroprotective function by evaluating neurons from the hippocampus in the I/R rat model. Hematoxylin and eosin staining revealed that I/R treatment reduced the survival of neurons in the hippocampus, and GJ treatment dose-dependently rescued this phenotype (Figure 2, p < 0.01). These suggest that GJ attenuates neurological loss in I/R rat hippocampus.

### 3.3. GJ represses I/R rat ischemic tissues apoptosis

**Figure 3.**
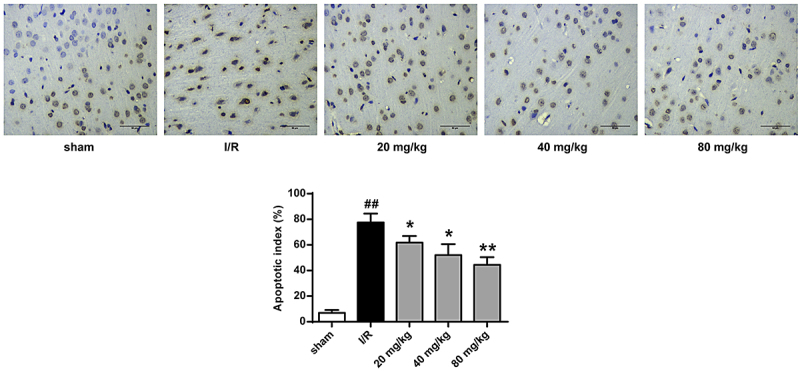
GJ represses apoptosis of ischemic tissues in I/R rats. MCAO/R rats were constructed and treated with GJ at indicated doses (n = 6). The apoptosis of cerebral cortex tissues was analyzed by TUNEL staining. * *P* < 0.05, ** *P* < 0.01, ^##^ *P* < 0.01. Data are presented as mean ± SD.

**Figure 4.**
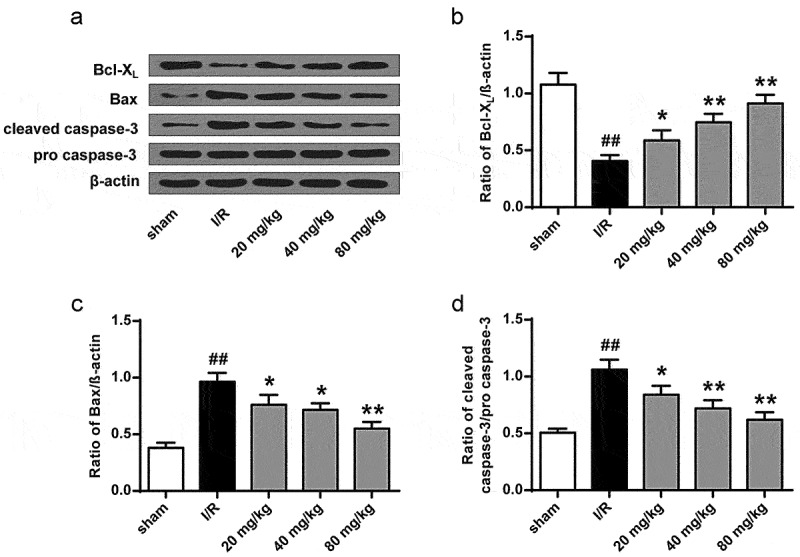
GJ regulates apoptotic proteins in I/R rat ischemic tissues. (a-d) MCAO/R rats were constructed and treated with GJ at indicated doses (n = 6). β-actin, cleaved caspase-3, Bax, and Bcl-X_L_ levels was analyzed by Western blot. * *P* < 0.05, ** *P* < 0.01, ^##^ *P* < 0.01. Data are presented as mean ± SD.

We then assessed the role of GJ in modulating apoptosis of ischemic tissues in I/R rats using TUNEL staining. The number of TUNEL positive cells was increased in the cerebral cortex tissues of I/R rats, whereas GJ treatment blocked this elevation (*P* < 0.01, Figure 3). In addition, the levels of pro-apoptotic cleaved caspase-3 and Bax expression were enhanced, while the level of anti-apoptotic Bcl-X_L_ expression was reduced in the I/R rats, and these changes were reversed by GJ treatment (Figure 4, p < 0.01). Altogether, these indicate that GJ represses the apoptosis of ischemic tissues in I/R rats.

### 3.4. GJ inhibits oxidative stress and inflammation in I/R rats

**Figure 5.**
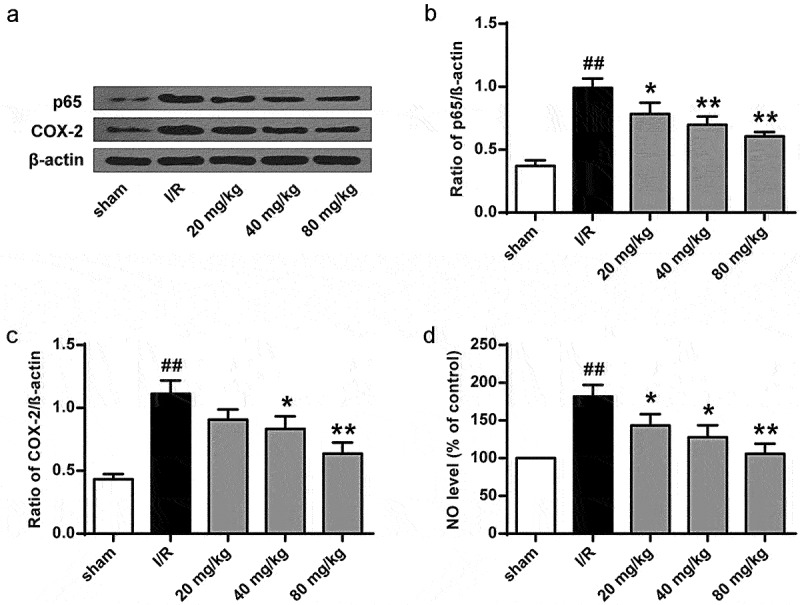
GJ inhibits inflammation in I/R rats. (a-d) MCAO/R rats were constructed and treated with GJ at indicated doses (n = 6). (a-c) NF-κB (p-p65), COX-2, and β-actin expression was measured. (d) NO levels were analyzed in rats. * *P* < 0.05, ** *P* < 0.01, ^##^ *P* < 0.01. Data are presented as mean ± SD.

Next, to further explore the anti-inflammatory effect of GJ, we measured p-p65 of NF-κB and COX-2 levels in I/R rat brain tissues. Significantly, I/R treatment enhanced p-p65 and COX-2 levels in rats, and these enhancements were reduced by GJ treatment in a dose-dependent manner (*P* < 0.01, Figure 5(a-c)). Meanwhile, the NO level in I/R rat brain was reduced by GJ treatment in a dose-dependent manner (*P* < 0.01, Figure 5(d)). Together, these data suggest that GJ treatment reduces inflammation in I/R rats.

**Figure 6.**
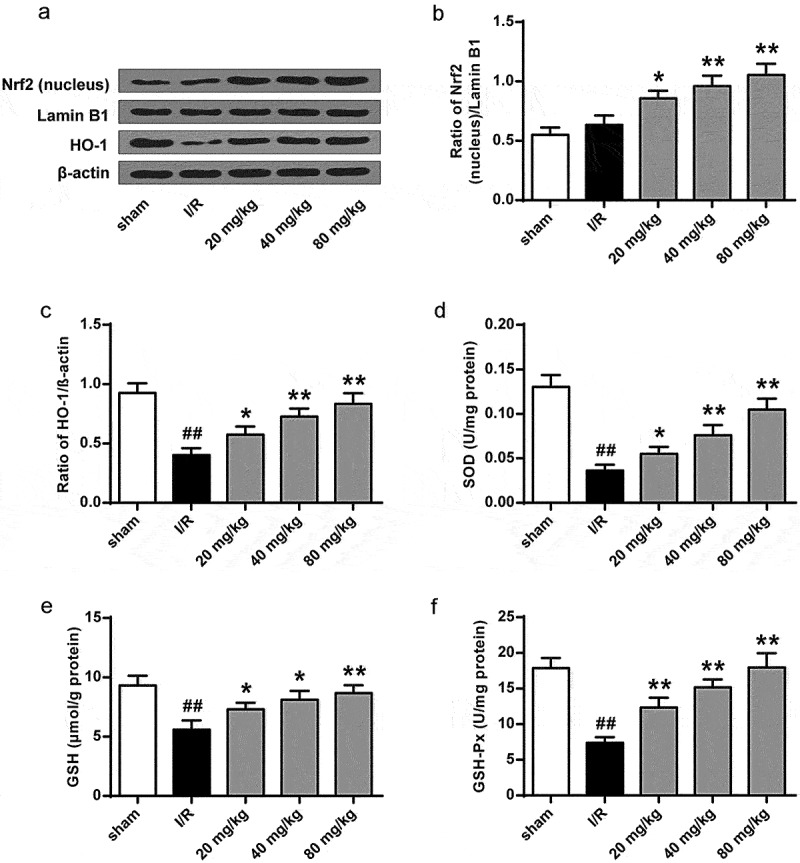
GJ inhibits oxidative stress in I/R rats. (a-f) MCAO/R rats were constructed and treated with GJ at indicated doses (n = 6). (a-c) Nucleus Nrf2, Lamin B1, HO-1, and β-actin expression was measured by Western blot. (d-f) SOD and GSH-Px activities and GSH content were analyzed. * *P* < 0.05, ** *P* < 0.01, ^##^ *P* < 0.01. Data are presented as mean ± SD.

To further investigate GJ’s antioxidant function in I/R rats, we examined Nrf2 nuclear translocation and HO-1 expression. The results showed that Nrf2 nuclear translocation and HO-1 expression were significantly enhanced by GJ in a dose-dependent manner in I/R rats (*P* < 0.01, Figure 6(a-c)). Meanwhile, the activities of antioxidant enzymes SOD (Figure 6(d)) and GSH-Px (Figure 6(f)) and the level of GSH (Figure 6(e)) were reduced by I/R treatment in rats (*P* < 0.01), while GJ treatment dose-dependently reversed these reductions. These results indicate that GJ exerts an antioxidant role in I/R rats.

### 3.5. GJ decreases lipid peroxidation in I/R rats

**Figure 7.**
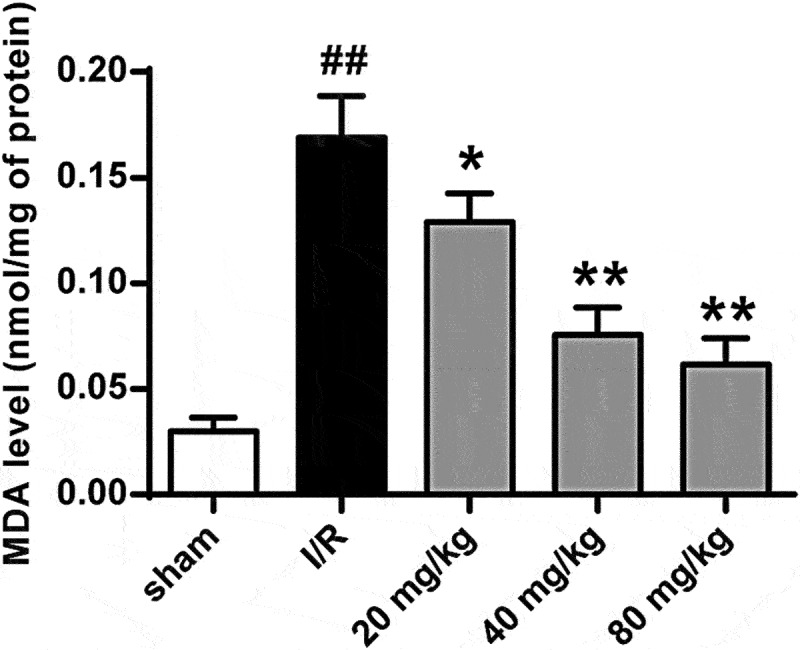
GJ decreases lipid peroxidation in I/R rats. MCAO/R rats were constructed and treated with GJ at indicated doses (n = 6). MDA levels were analyzed in rats. * *P* < 0.05, ** *P* < 0.01, ^##^ *P* < 0.01. Data are presented as mean ± SD.

To further investigate GJ’s function in I/R injury-induced lipid peroxidation, we measured MDA levels in I/R rat brain tissues. Our data showed that I/R significantly increased MDA level in the rats, and GJ treatment dose-dependently reduced this enhancement (*P* < 0.01, Figure 7). These data suggest that GJ represses lipid peroxidation in I/R rats.

## 4. Discussion

CIR injury is one of the most severe cerebrovascular disorders [4] and is correlated with inflammatory response, oxidative stress, and hypoxia [6]. Recently, natural compounds of traditional Chinese herbs have been used to treat cerebrovascular diseases [9–11]. GJ, a principal constituent of *S. Chinensis*, has presented multiple biomedical activities, including anti-inflammatory effects and vascular relaxation [19–21]. In this study, we found that GJ treatment attenuates CIR injury by inducing anti-apoptotic, anti-inflammatory, and antioxidant effects in rats.

In recent years, natural compounds have been identified to modulate CIR injury. For instance, hexahydrocurcumin prevents CIR injury by inhibiting oxidant stress and inflammation in the stroke rat model [27]. YiQi Tongluo Granule attenuates CIR injury through regulating CaMKII and GluN2B via NMDAR-ERK1/2 signaling in rats [28]. Trametenolic acid B reduces CIR injury by modulating the microRNA-10a/PI3K/Akt/mTOR axis [29]. Ginkgo diterpene lactones repress CIR-induced inflammation response via the TLR4/NF-κB signaling in rat astrocytes [30]. Qingkailing injection protects against CIR injury and regulates the AMPK/NLRP3 inflammation signaling [31]. Here, we first identified that GI could relieve CIR-induced neuronal injury and neurological loss of the hippocampus in I/R rats, suggesting that GI protects against CIR-related brain dysfunction. Our study provides valuable evidence for GI’s function in cerebrovascular disease development.

Inhibiting inflammation, apoptosis, oxidative stress, and lipid peroxidation can attenuate CIR injury. For instance, RTN1-C interferes CIR injury by regulating apoptosis and ER stress processes [22]. Trim47 inhibits CIR injury by modulating inflammation and apoptosis [32]. DUSP14 relieves CIR injury through inhibiting apoptosis and inflammation by activating Nrf-2 [33]. MicroRNA-106b-5p reduces CIR injury by repressing apoptosis and oxidative stress in rats [34]. Nodal reduces CIR injury by inhibiting inflammation and oxidative stress [35]. Theaflavin inhibits CIR injury by suppressing oxidative stress [36]. Biochanin A induces neuroprotection against CIR injury by inhibiting oxidative stress and inflammation signaling in rats [37]. Monosialotetrahexosylganglioside protects CIR injury by inhibiting lipid peroxidation [38]. Moreover, as a natural component of *S. Chinensis*, GJ is well known to have an anti-cancer effect [39]. It has been found that GJ is able to inhibit inflammation and oxidative stress [20,40,41]. Our study showed that GJ inhibits inflammation, apoptosis, oxidative stress, and lipid peroxidation in I/R rats. Our study has some limitations. First, we mainly used young animals instead of old animals. Our results need to be further evaluated in old animals in the future [42]. In addition, our study did not explore the effect of GJ treatment on transport proteins [43]. Moreover, the effects of GJ treatment on some key immune cells, such as leukocytes and neutrophils, will be explored in future studies [44].

## 5. Conclusions

In conclusion, we identified that GJ attenuates CIR injury by inducing anti-apoptotic, anti-inflammatory, and antioxidant effects *in vivo*. This study provides new insight into GJ’s function in stroke-related CIR injury and indicates that GJ may serve as a potential candidate for stroke-related CIR injury treatment.

## Data Availability

The data that support the findings of this study are available on request from the corresponding author.
